# Gingival varices treated with monoethanolamine oleate: A rare case report

**DOI:** 10.4317/jced.53893

**Published:** 2018-02-01

**Authors:** Sarah Campos-de Sales, Camila-Nazaré-Alves-de Oliveira Kato, Mariana-Saturnino de Noronha, Wagner-Henriques Castro, Ricardo-Alves Mesquita

**Affiliations:** 1 DDS, Department of Oral Surgery and Pathology, School of Dentistry, Universidade Federal of Minas Gerais, Belo Horizonte, Brasil; 2 DDS, Msc student, Department of Oral Surgery and Pathology, School of Dentistry, Universidade Federal of Minas Gerais, Belo Horizonte, Brasil; 3 DDS, Msc student, Department of Oral Surgery and Pathology, School of Dentistry, Universidade Federal of Minas Gerais, Belo Horizonte, Brasil; 4 Professor, Msc, PhD, Department of Oral Surgery and Pathology, School of Dentistry, Universidade Federal of Minas Gerais, Belo Horizonte, Brasil

## Abstract

Varices are benign blood vessel lesions that are common in the head and neck regions. The aim of this case report is to highlight an uncommon case of gingival varices and its diagnosis and management. This is the second time that a case of varices has been reported at this site. Monoethanolamine oleate (MO) at a 2.5% concentration was used as the treatment. A 66-year-old woman presented spontaneous gingival bleeding in the region of the mandibular first and second left molars. A macula and gingival enlargement on the interproximal papillae were observed. No bleeding was observed during the oral examination. The clinical diagnosis was varices. The patient was given two sessions of sclerotherapy with 2.5% MO applied to the lesion, with 15 days between applications. The lesion showed total clinical resolution, and the patient is in follow-up. This paper reports a rare case of varices in the gingival mandible, with the diagnosis based on the patient’s age, time evolution of the lesion’s, and its clinical characteristics. The concentration of 2.5% MO is safe and efficient, a conservative treatment, and easy to apply.

## Introduction

Vascular anomalies (VAs) are benign lesions that affect blood vessels. For the most part, these anomalies affect the head and neck, the oral cavity, and the upper aerodigestive tract (1). Mulliken and Glowacki (2) classified VA into hemangiomas and vascular malformations. As many types of VAs have been described, some are similar, and the terminology is frequently imprecise. In the current classification, two groups of lesions are known: tumors and malformations (3). The malformations are subdivided into low-flow (venous, lymphatic, and capillary) and high-flow (3,4). Varices represent acquired anomalies of extremely dilated local dermal or submucosal veins.

Studies of oral lesion prevalence have indicated that 7-9% are varices (5,6). A descriptive analysis of the prevalence of 154 VAs showed oral varices as the most frequent vascular lesion (65.6%) (7). Clinically, varices occur as red to purple papulae, nodules, or ridges commonly found on the tongue, lip, or cheeks, but they are rare in the gingiva (8,9). Diagnosis depends on the clinical manifestations, the age of the patient, etiologic factors, and the anatomical distribution of the disease (9). Cryosurgery and sclerotherapy have been used in the treatment of oral varices (7,8,10,11).

Considering the rare occurrence of and the difficulty of managing varices in the gingiva, the aim of this case report is to describe gingival varices treated with sclerotherapy. This kind of lesion was reported before (12) but is still very rare; this is only the second report.

## Case report

A 66-year-old white woman was referred to the Oral Medicine Service, School of Dentistry, Universidade Federal Minas Gerais, Brazil, for evaluation of spontaneous bleeding between the mandibular first and second left molars. Her medical history revealed controlled systemic arterial hypertension.

**Figure 1:**
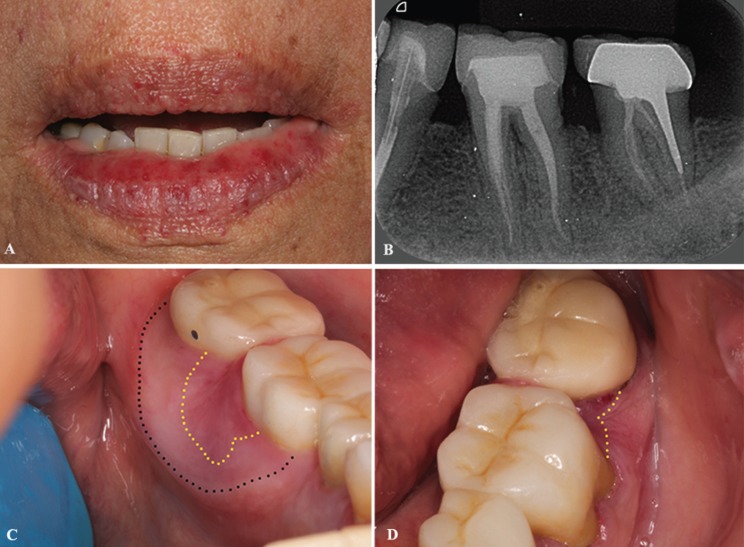
A) Extraoral view of the patient, multiple punctate erythematous macules on the upper and lower lips are noted. B) Periapical radiograph demonstrated endodontic treatments in the mandibular second left premolar, first and second left molars, but no changes associated with the alveolar bone. C,D) Clinical presentation of the lesion, red macula on lingual and vestibule gingiva (yellow dotted), and gingival swelling on lingual (black dotted). C) Lingual view; B) Vestibular view.

The patient reported that the bleeding had been present for 3 years. She also had several episodes of difficulty containing the bleeding, which she described as “filling the mouth”. A clinical extraoral exam showed multiple punctate erythematous papulae on the upper and lower lips (Fig. 1A). A periapical radiograph showed evidence of endodontic treatment on the mandibular second left premolar and first and second left molars, but no changes were associated with the alveolar bone (Fig. 1B). An intraoral examination found, near the mandibular left first and second molars, an erythematous and irregular macula in the gingival region that was approximately 10 mm from the lingual area and 3 mm from the cervical vestibular area, as well as tissue enlargement on the interproximal papillae. We observed a swelling in the lingual area, which was not involved in the lesion (Fig. 1C,D). No bleeding was observed during oral examination. The international normalized ratio (INR) for prothrombin times and the complete blood count (CBC) showed regular values.

**Figure 2:**
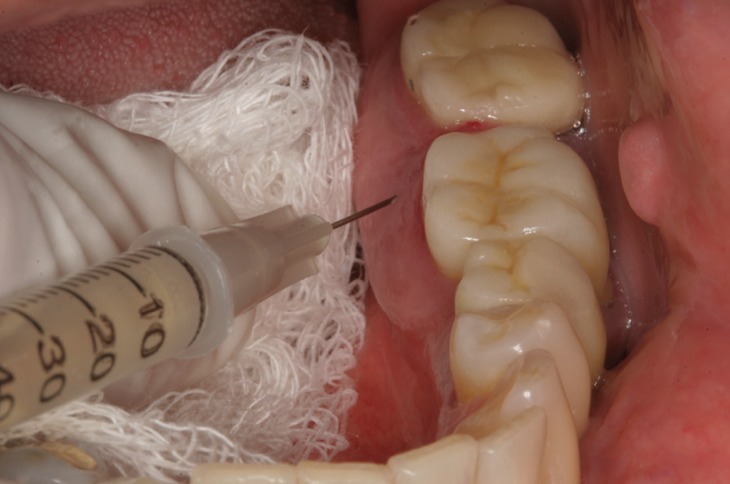
Treatment with 0.1 mL of the 2.5% monoethanolamine oleate of gingival.

A diagnosis of varices was made for the red macula. The patient was informed, and a written informed consent was obtained prior to the treatment. The proposed treatment was sclerotherapy with 0.1 ml of 2.5% monoethanolamine oleate (MO), given through intraluminal injection in the mandibular gingival lingual region, near the first and second left molars (Fig. 2). The patient was treated with two sessions of sclerotherapy, with a 15-day interval between applications (11).

**Figure 3:**
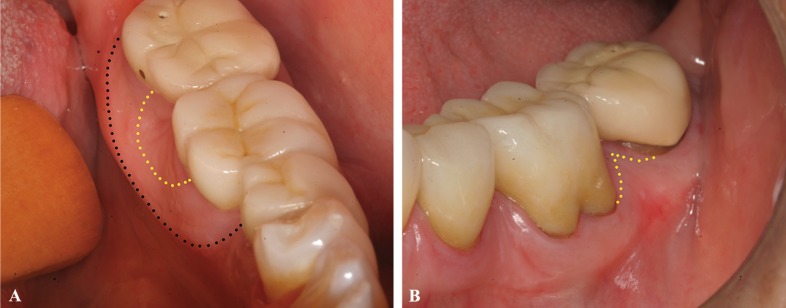
Following-up of 6 months. Total clinical resolution of the lesion (yellow dotted) and remains swelling in the lingual area (black dotted) A: Lingual view B: Vestibular view.

The varices regressed after two therapy sessions, and according to the patient, the spontaneous bleeding has stopped (Fig. 3). Lingual swelling remains with no alterations; it was diagnosed as hypertrophic gingiva. No recurrence was observed after 18 months.

## Discussion

The incidence of sublingual varices has been shown to increase with age (8). The pathogenesis of the varices is associated with age-related degeneration, which occurs loss of the conjunctive tissue tonus that supports vessel tonus (13). The possible connection between cardiovascular diseases and varices has been discussed, and it appears that sublingual varices are significantly associated with age, smoking, and cardiovascular diseases, although the authors could not explain the relationship (8,14). Although varices are frequent vascular diseases, they are not included in the classification of VAs (3,4).

The ventral surface of the tongue is a common site of oral varices. The floor of the mouth, the lips, and the buccal mucosa are less common sites (6). This study reports a rare case of gingival varices in the mandible. In 1973, another case of oral varices was described, which the patient was 63-year-old, like the current case, and had a large, red, asymptomatic nodule of 5-6 years’ evolution on the mandibular gingiva. The treatment was surgical excision. Through a histopathological examination, a diagnosis of varices was given (12).

Hemangioma, vascular malformation, and oral pyogenic granuloma are differential diagnoses of oral varices. Hemangioma is a proliferation of endothelial cells common in infancy, which displays a rapid growth phase followed by a gradual involution. Vascular malformations are structural abnormalities of the blood vessels without endothelial proliferation. These conditions are present at birth and persist throughout life, while the pyogenic granuloma is an exaggerated soft tissue response and vascular proliferation resulting from chronic low-grade irritation. It is most common in children and young adults (15). In contrast, varices are benign, acquired vascular lesions and common in older people (7,8).

In most cases of oral varices, the history of the disease and the clinical examination are sufficient to establish the diagnosis (10). In the current study, the diagnosis was based on the patient’s age, time evolution of lesion, and lack of spontaneous involution of the lesion, as well as the observation of erythematous areas on the lips.

Varices are generally asymptomatic and require not treatment (7). In this case, the patient presented several episodes of difficulty containing the bleeding that affected her quality of life. Therefore, treatment was necessary. Sclerotherapy with MO is a treatment for VA located in various parts of the body. Treatment with MO is painless and causes no fibrosis, no burning, tissue loss, or bleeding; it is also low-cost, easy to apply, and a conservative treatment (11). These features make MO a good choice for management of gingival diseases.

This paper reports a rare case of varices in the gingival mandible, with a diagnosis based on the patient’s age, time of lesion evolution, and clinical characteristics. Treatment with 2.5% MO was a safe and efficient choice.
